# Recurrent Lower Gastrointestinal Bleeding: Ileal GIST Diagnosed by Video Capsule Endoscopy—A Case Report and Literature Review

**DOI:** 10.1155/2013/285457

**Published:** 2013-08-21

**Authors:** Jie Ling, Marie Lamsen, Roger Coron, Danila Deliana, Sabah Siddiqui, Madhu Rangraj, Stephen Jesmajian

**Affiliations:** ^1^Department of Internal Medicine, Sound Shore Medical Center, 16 Guion Place, New Rochelle, NY 10801, USA; ^2^Department of Gastroenterology, Sound Shore Medical Center, 16 Guion Place, New Rochelle, NY 10801, USA; ^3^Department of Surgery, Sound Shore Medical Center, 16 Guion Place, New Rochelle, NY 10801, USA

## Abstract

*Introduction*. Gastrointestinal stromal tumor (GIST) in the ileum is an extremely rare cause of recurrent lower gastrointestinal bleeding (GIB). *Case Report*. An 89-year-old man was admitted with melana. He had extensive PMH of CAD post-CABG/AICD, AAA repair, chronic anemia, myelodysplastic syndrome, lung cancer after resection, and recurrent GIB. Prior EGDs, colonoscopies, and upper device-assisted enteroscopy showed duodenal ulcer, A-V malformation s/p cauterization, and angioectasia. On admission, Hb was 6.0 g/dL. An endoscopic capsule study showed an ulcerated tumor in the ileum. CT showed no distant metastasis. The lesion was resected successfully and confirmed as a high-grade GIST. The patient was discharged with no further bleeding. *Discussion*. Early diagnosis for patients with ileal GIST is often challenging. Video capsule endoscopy and double balloon enteroscopy could be useful diagnostic tools. Surgical removal is the first line for a resectable GIST. Imatinib has become the standard therapy. *Conclusion*. This is a unique case of an ileal GIST in a patient with recurrent GIB which was diagnosed by video capsule. Complicated medical comorbidities often lead to a significant delay in diagnosis. Therefore, we recommend that if GIB does not resolve after appropriate treatments for known causes, the alternative diagnosis for occult GIB must be considered, including malignancy such as GIST.

## 1. Introduction

Lower gastrointestinal bleeding (GIB) is the common cause of hospitalization, often involving bleeding from colitis, hemorrhoids, cancer, and vascular anomalies [1]. However, GIST is an extremely rare cause of lower GIB.

*GISTs* are infrequent neoplasms with a reported annual incidence of 6.8 per million in the USA, more commonly occurring in middle-aged and older people with approximately equal sex distribution [2]. However, they are the most common mesenchymal malignancas of the gastrointestinal tract, which were first described by Mazur MT and Clark HB in 1983 [3]. A GIST can be located anywhere in the gastrointestinal tract, the most common sites being the stomach (63%), followed by small intestine (23%), colorectal (5%), and esophagus (1.6%) [4]. Histologically, they are mesenchymal spindle cells and immunohistochemically positive for tyrosine kinase receptor CD 117 (c-KIT), related tyrosine kinase receptor PDGFR (platelet-derived growth factor receptor *α*, a KIT), and CD34 expression [5]. KIT has been demonstrated as a very specific and sensitive marker to mesenchymal tumors in the GI tract and around 95% of GISTs express KIT.

Depending on the size, location, and the presence of mucosal ulceration, the clinical presentation of GIST varies significantly including bleeding, abdominal pain, dyspepsia intestinal obstruction, and so forth. Approximately half of individuals with GIST present with anemia or anemia-associated symptoms due to mucosal ulceration of a tumor [6]. GISTs may also be discovered as an incidental finding during radiologic imaging, endoscopy, or abdominal surgery performed for other reasons. Patients with clinically malignant GISTs may present with disseminated disease. Metastases occur primarily in the liver and abdominal cavity. Metastases to the lung, bone, lymph nodes, skin, or soft tissues are rare and generally only seen in the setting of very late-stage disease. Overall 5-year survival is about 35% [7].

We present a GIB case of an elderly patient with a GIST arising from the mid of ileum, who had extensive past medical history including coronary artery disease, abdominal aortic aneurysm repair, chronic anemia secondary to myelodysplastic syndrome, lung cancer after resection, gastritis with aspirin use, AV malformation, and superficial duodenal ulcer, which could all be contributing factors for anemia and GIB. The recurrent lower GIB from GIST has been reported infrequently. To the best of our knowledge, no previous case with such extensive medical history has ever been reported.

## 2. Patient, Methods, and Results 

An 89-year-old man was admitted with dark melanotic stool for a few weeks. He had extensive PMH of CAD post-CABG (coronary artery bypass graft) and AICD/PPM (atrial/permanent pacemaker), AAA s/p surgical repair, chronic anemia secondary to MDS, lung cancer after resection, history of iron deficiency anemia, and 6-month recurrent lower GIb (hematochezia and melanotic stools). Before admission to our hospital, he had two prior hospitalizations in other institutions for acute or chronic anemia and hematochezia, respectively. Multiple EGDs, colonoscopies, upper device-assisted enteroscopy with fluoroscopy, incomplete capsule endoscopy (where the capsule got stuck in the stomach) were performed, which collectively showed gastritis, AV malformation s/p cauterization, nonbleeding superficial duodenal ulcer with a single angioectasia, and numerous small mouthed diverticula in recto-sigmoid colon. He was on aspirin, metoprolol, pravastatin, and epoetin alfa and received multiple blood transfusions in the past. Most recent transfusion was a week prior to admission. He had no drug allergies.

Clinical examination was unremarkable except for pallor and strongly positive guaiac test. Heart rate was 75 bpm (ventricular paced). Blood tests showed marked anemia with Hb of 6.0 g/dL (MCV 84 fL) with normal LFTs and clotting factors. Echo showed EF of 35% with aortic stenosis. 

**Figure 1:**
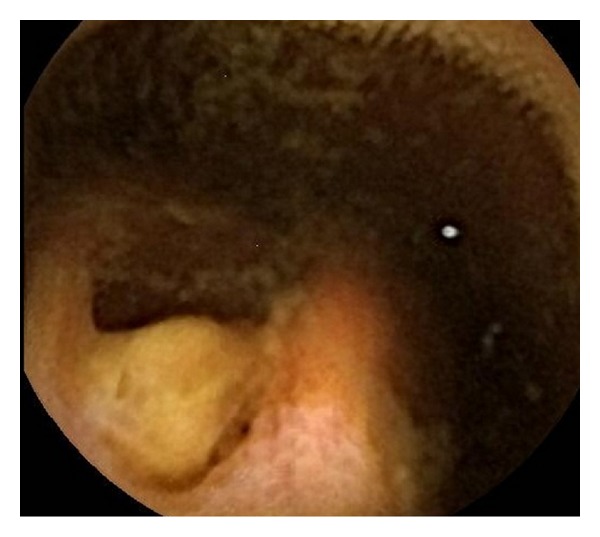
Capsule endoscopic finding: a tumor arising from small intestine with intraluminal growth with ulcerated surface.

**Figure 2:**
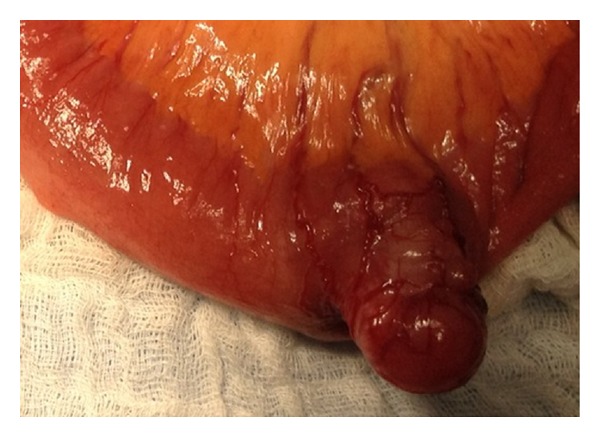
Intraoperative finding: a tumor arising from the ileum with extraluminal growth.

**Figure 3:**
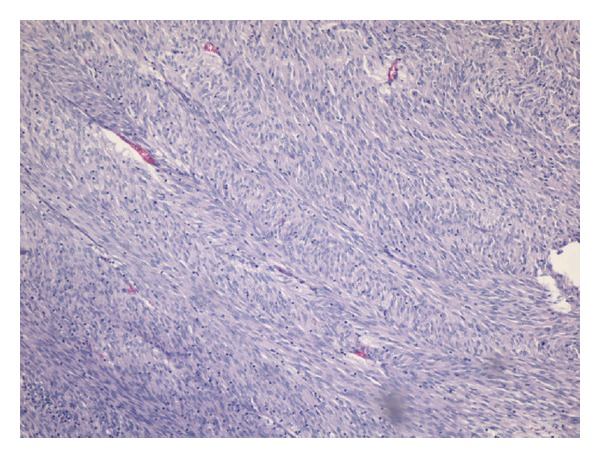
Microscopic finding: histological examination showing bundles of spindle cells with elongated nuclei and significant mitotic figures (H and E stain; 100x).

**Figure 4:**
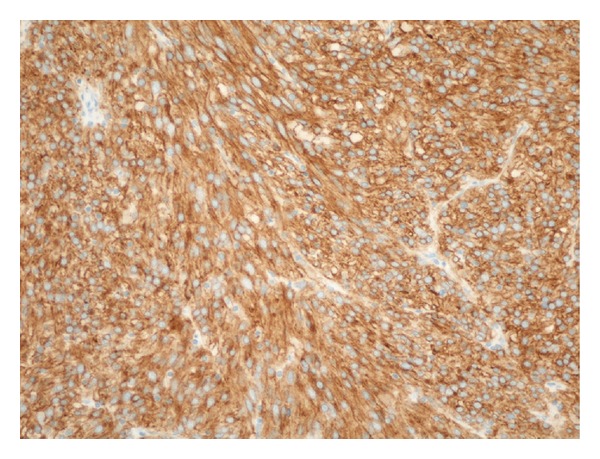
Microscopic finding: immunohistochemistry exhibiting a strong positive for CD117 (CD117 stain; 200x).

On the second day of admission, an endoscopic capsule study was performed which showed an ulcerated tumor in the small bowel with intraluminal growth with ulcerated surface (Figure 1), which was likely the source of the GIb. Preoperative CT showed no distal metastasis. On the third day of admission, an exploratory laparotomy was performed before the capsule passed out of the rectum, which revealed an irregular, firm mass, in total 4.5 × 3.2 × 2.5 cm, in midileum (Figure 2) with a 2 × 2 cm mass protruding from the antimesenteric wall. The lesion was resected with primary end-to-end anastomosis. Histology revealed a high-grade ulcerated GIST with classic spinal cells, clear resection margins, and no lymph node invasion (Figure 3). Immunohistochemistry was positive for c-kit (CD117) and CD34 immunomarkers (Figure 4). Ki67 proliferation marker labels 10% of the nuclei. Mitotic activity was 23/50 on high-power fields.

Though our patient could be a candidate for Imatinib treatment, given his age, preference, comorbidity, and possible side effects of Imatinib (especially severe fluid retention and congestive heart failure), we decided not to treat him with neoadjuvant Imatinib therapy. He made an uneventful recovery with no further bleeding and was discharged 10 days after admission. The patient has remained disease-free and has shown no further GIb for 2 months postoperatively.

## 3. Discussion

GIST is unusual cause of lower GIB, which is a common cause of hospitalization, often involving bleeding from diverticulosis, colitis, hemorrhoids, cancer, and polyps [1]. Tran et al. [2] reviewed 1,312 archival pathologic specimens of the small intestine in 10-year duration in search for malignant tumors and only 1 case of GIST tumor was found among 41 identified tumors. 

Though most GIST tumors lead to gross ulceration of the mucosa likely to be detected on endoscopic examination, similar to our patient, an accurate and early diagnosis for patients with ileal GIST is often challenging due to the diagnostic limitations. Hadithi [8] reports that the judicious use of video capsule endoscopy (VCE) and double balloon enteroscopy (DBE) could be useful diagnostic tools with detection rates of 80% and 60% for VCE and DBE, respectively. Other diagnostic methods include contrast-enhanced computed tomography (CT), CT angiograph, and Meckel's scan if Meckel's diverticulum is thought to be involved and diagnostic laparoscopy. 

From a literature search, the majority of patients diagnosed with GIST are in the 40 to 80 year range [4]. Surprisingly, no case report with ileal GIST involved extensive and coexisting PMH. Our report highlights an extremely rare case of ileal GIST with recurrent GIb with complicated PMH such as CAD, AAA, MDS, lung cancer, gastritis with aspirin use, gastric ulcer disease, and A-V malformation. These coexisting confounding factors for GIb could lead to diagnostic difficulties and a significant delay in diagnosis. In fact, it took a total of 6 months to diagnose the source of bleeding after he had multiple EGDs, colonoscopies, and numerous blood transfusions. Based on our experience, if GIb does not resolve after appropriate treatments for known causes, the alternative cause for occult GIb must be considered including malignancy such as GIST.

In terms of the treatment for GIST, surgical removal is often considered as the first-line for a resectable GIST [6]. Radiation and cytotoxic chemotherapy are ineffective in GISTs and are only used in refractory disease for palliative purposes. Imatinib mesylate, a targeted therapy as tyrosine kinase inhibitor of c-KIT and PDGFR-a, has become the standard therapy not only for recurrent or metastatic GIST, but also for the adjuvant treatment in adult patients following complete resection of KIT-positive GIST [9, 10].

The prognosis of GIST primarily correlates with tumor size, mitotic index, and location. The National Institutes of Health Consensus Workshop for GISTs proposed a risk stratification schema based on size and mitotic count in the hope of better defining groups of patients expected to exhibit an aggressive clinical course [11]. It was believed that gastric GISTs are less aggressive than small intestine GIST. In addition, many other independent predictors of survival have been described [2], including older age, black race, advanced stage, no surgical intervention or incomplete resection, and high Ki-67 count [12]. In our case, based on Fletcher's classification, the tumor belongs to the high-risk category given high mitotic activity of 23/50 HPF (>10/50 HPF). However, treatment with neoadjuvant Imatinib therapy was not recommended, given patient's age, preference, comorbidity, and possible side effects of Imatinib (especially severe fluid retention and congestive heart failure).

In conclusion, we presented a unique case of an ileal GIST presented with recurrent GIB and diagnosed by capsule and confirmed pathologically. Based on our literature search, only a few cases of recurrent GIB were due to ileal GIST in patients older than 16 years [13–15]. To the best of our knowledge, none of them had extensive and complicated PMH, which could all be possible sources of GIB and thus lead to significant delay in diagnosis. Therefore, we recommended that if GIB does not resolve after appropriate treatments for known causes, the alternative causes for occult GIB must be considered including malignancy such as GIST.

## References

[1] Zhao Y, Encinosa W (2009). Hospitalizations for gastrointestinal bleeding in 1998 and 2006. *Statistical Brief*.

[2] Tran T, Davila JA, El-Serag HB (2005). The epidemiology of malignant gastrointestinal stromal tumors: an analysis of 1,458 cases from 1992 to 2000. *American Journal of Gastroenterology*.

[3] Mazur MT, Clark HB (1983). Gastric stromal tumors. Reappraisal of histogenesis. *American Journal of Surgical Pathology*.

[4] Mucciarini C, Rossi G, Bertolini F (2007). Incidence and clinicopathologic features of gastrointestinal stromal tumors. A population-based study. *BMC Cancer*.

[5] Liegl B, Hornick JL, Lazar AJF (2009). Contemporary pathology of gastrointestinal stromal tumors. *Hematology/Oncology Clinics of North America*.

[6] Hueman MT, Schulick RD (2008). Management of gastrointestinal stromal tumors. *Surgical Clinics of North America*.

[7] DeMatteo RP, Lewis JJ, Leung D, Mudan SS, Woodruff JM, Brennan MF (2000). Two hundred gastrointestinal stromal tumors: recurrence patterns and prognostic factors for survival. *Annals of Surgery*.

[8] Hadithi M, Heine GD, Jacobs MA, van Bodegraven AA, Mulder CJ (2006). A prospective study comparing video capsule endoscopy with double-balloon enteroscopy in patients with obscure gastrointestinal bleeding. *American Journal of Gastroenterology*.

[9] http://www.pharma.us.novartis.com/product/pi/pdf/gleevec_tabs.pdf.

[10] Eisenberg BL, Harris J, Blanke CD (2009). Phase II trial of neoadjuvant/adjuvant imatinib mesylate (IM) for advanced primary and metastatic/recurrent operable gastrointestinal stromal tumor (GIST): early results of RTOG 0132/ACRIN 6665. *Journal of Surgical Oncology*.

[11] Fletcher CD, Berman JJ, Corless C (2002). Diagnosis of gastrointestinal stromal tumors: a consensus approach. *Human Pathology*.

[12] Saponara M, di Battista M, Lolli C (2009). Evaluation of Ki-67 in gastrointestinal stromal tumor (GIST). *Journal of Clinical Oncology*.

[13] van Loo S, van Thielen J, Cools P (2010). Gastrointestinal bleeding caused by a GIST of Meckel’s diverticulum—a case report. *Acta Chirurgica Belgica*.

[14] Worley TA, Abadin SS, Revesz E, Salti GI (2010). Gastrointestinal stromal tumor with hemoperitoneum masquerading as appendicitis. *International Surgery*.

[15] Abbassi-Ghadi N, Bartlam K, Rasheed S, Holme T (2009). A case of obscure gastrointestinal bleeding in a teenager. *Annals of the Royal College of Surgeons of England*.

